# Inter and intra-tumor somatostatin receptor 2 heterogeneity influences peptide receptor radionuclide therapy response

**DOI:** 10.7150/thno.51215

**Published:** 2021-01-01

**Authors:** Danny Feijtel, Gabriela N. Doeswijk, Nicole S. Verkaik, Joost C. Haeck, Daniela Chicco, Carmelina Angotti, Mark W. Konijnenberg, Marion de Jong, Julie Nonnekens

**Affiliations:** 1 Department of Radiology and Nuclear Medicine, Erasmus MC Rotterdam, The Netherlands; 2 Department of Molecular Genetics, Erasmus MC Rotterdam, The Netherlands; 3 Oncode Institute, Erasmus MC Rotterdam, The Netherlands; 4 Advanced Accelerator Applications SA, a Novartis Company, Erasmus MC Rotterdam, The Netherlands

**Keywords:** Peptide receptor radionuclide therapy, neuroendocrine tumors, somatostatin receptor subtype 2, radiobiology, DNA damage response

## Abstract

Patients with neuroendocrine tumors (NETs) can be treated with peptide receptor radionuclide therapy (PRRT). Here, the somatostatin analogue octreotate radiolabeled with lutetium-177 is targeted to NET cells by binding to the somatostatin receptor subtype 2 (SST_2_). During radioactive decay, DNA damage is induced, leading to NET cell death. Although the therapy proves to be effective, mortality rates remain high. To appropriately select more optimal treatment strategies, it is essential to first better understand the radiobiological responses of tumor cells to PRRT.

**Methods:** We analyzed PRRT induced radiobiological responses in SST_2_ expressing cells and xenografted mice using SPECT/MRI scanning and histological and molecular analyses. We measured [^177^Lu]Lu-DOTA-TATE uptake and performed analyses to visualize induction of DNA damage, cell death and other cellular characteristics.

**Results:** The highest accumulation of radioactivity was measured in the tumor and kidneys. PRRT induced DNA damage signaling and repair in a time-dependent manner. We observed intra-tumor heterogeneity of DNA damage and apoptosis, which was not attributed to proliferation or bioavailability. We found a strong correlation between high DNA damage levels and high SST_2_ expression. PRRT elicited a different therapeutic response between models with different SST_2_ expression levels. Heterogeneous SST_2_ expression levels were also confirmed in patient NETs.

**Conclusion:** Heterogeneous SST_2_ expression levels within NETs cause differentially induced DNA damage levels, influence recurrent tumor phenotypes and impact the therapeutic response in different models and potentially in patients. Our results contribute to a better understanding of PRRT effects, which might impact future therapeutic outcome of NET patients.

## Introduction

Neuroendocrine tumors (NETs) form a heterogeneous group of tumors, nonetheless 70-100% of differentiated NETs highly express the somatostatin receptor subtype 2 (SST_2_) on their cell membrane 1. These receptors can be targeted with radiolabeled somatostatin analogues, such as [Tyr^3^]octreotate, for diagnostics or therapy 2, 3. Peptide receptor radionuclide therapy (PRRT) using [Tyr^3^]octreotate labeled with the β-particle emitter lutetium-177 ([^177^Lu]Lu-DOTA-[Tyr^3^]octreotate or [^177^Lu]Lu-DOTA-TATE) has proven to be an effective therapy for patients with non-operable or metastatic SST_2_ positive NETs in terms of improving progression free survival (PFS) and quality of life 4, 5.

During PRRT, [^177^Lu]Lu-DOTA-TATE is targeted to NETs via SST_2_ binding and will deliver a cytotoxic radiation dose to the cancer cells 6. PRRT outperforms other available treatments for metastasized NETs on various levels, but unfortunately still lacks the efficacy for complete remission in the majority of patients 5. Increasing the therapeutic efficacy might be accomplished by changing the treatment planning (time interval or dosing), by developing new targeting biomolecules 7, 8, by using more powerful radionuclides 9 or by combining PRRT with other treatments, such as DNA damage repair modulating compounds 10.

Before the best PRRT optimization strategy can be determined, it is essential to first better understand the biological effects of ionizing radiation, i.e. radiobiology. However, until now little information is available 11. In sharp contrast, radiobiological principles of external beam radiotherapy (EBRT) have been studied for decades and these studies have significantly contributed to breakthroughs in improving effectiveness of EBRT 12. Unfortunately, due to fundamental differences in radiation qualities between EBRT and PRRT, such as dose-rate and continuous radiation versus single or fractionated doses, extrapolation of radiobiological knowledge is not straightforward 13. To make calculated decisions for therapy optimization, first a better understanding of the radiobiological effects of PRRT has to be obtained 11. For this purpose, we dissected radiobiological responses of PRRT in different *in vivo* and *in vitro* SST_2_ expressing models. Our analyses showed that important biological parameters, such as SST_2_ expression levels, can differ within and between models and human NET samples. Furthermore, we have demonstrated that differential SST_2_ expression levels are an important determinant for some radiobiological effects of PRRT in NET cells.

## Methods

### Cell culture and *in vitro* treatment

NCI-H69 cells (*ATCC*) were cultured in Rosewell Park Medium Institute 1638 medium (RPMI-1638) (Sigma-Aldrich) supplemented with penicillin (50 units/mL), streptomycin (50 µg/mL) (Pen/Strep) and 10% fetal calf serum (FCS). CA20948 14 cells were cultured in Dulbecco's Modified Eagle's Medium (DMEM) (Gibco) supplemented with Pen/Strep and 10% FCS. Cells were cultured at 37 ^o^C and 5% CO_2_.

The molar activity of [^177^Lu]Lu-DOTA-TATE used *in vitro* was 37 MBq/µg with a purity > 95%. Both CA20948 and NCI-H69 cells were incubated with 1 MBq/mL [^177^Lu]Lu-DOTA-TATE for 4 h at 37 ^o^C. NCI-H69 cells were spun down on a glass slide using a cytospin centrifuge (Rotofix 32A, Hettich) for 6 minutes at 347 g at room temperature (RT). CA20948 cells were washed and fixed on coverslips. Fixation was done using 2% paraformaldehyde (PFA) for 20 minutes at RT and samples were stained according to protocol (see below).

### Animal experimental conditions

Animal experiments were approved by the Animal Welfare Committee of the Erasmus MC and were conducted in accordance with European guidelines. Animal experiments included a time series for analyses of radiobiological parameters in NCI-H69 xenografts and survival cohorts with NCI-H69 or CA20948 xenografts. For both animal experiments 30 MBq/0.5 µg [^177^Lu]Lu-DOTA-TATE was labeled as previously described with a purity of > 95% 15. All animals received a single injection of 30 MBq [^177^Lu]Lu-DOTA-TATE.

### Human NET tissue samples

Pancreatic NET tissue was obtained from patients undergoing surgery. Tissue was obtained according to the code of proper secondary use of human tissue in the Netherlands established by the Dutch Federation of Medical Scientific Societies and approved by the local Medical Ethical committees. Specimens were coded anonymously.

### *In vivo* treatment for radiobiological analysis

BALB-c/nude mice were engrafted subcutaneously with 5 x 10^6^ NCI-H69 cells in 200 µl Hanks Balanced Salt Solution (HBSS) (Gibco, 14065056) containing 33,3% Matrigel (Corning, 354248). Tumor volumes were measured every 2 days by palpation and at a tumor size of 369 ± 203 mm^3^ mice were injected intravenously: 100 µL (40 g/L) gelofusine (Braun Medical) and after 2 minutes 30 MBq/0,5 µg [^177^Lu]Lu-DOTA-TATE in 0,1% Bovine serum albumin (BSA) in phosphate buffered saline (PBS) (Sigma, D8537-500 mL) (n = 4 per group). Mice were sacrificed by cervical dislocation and analyzed at 1 h, 1, 2, 3, 4, 5, 7, 9, 11 and 14 days post injection (p.i.). Organs were put in a gamma-counter for measurement of radioactive uptake and then fixed in formalin for 1 day at RT and stored in 70% ethanol until they were embedded in paraffin. Uninjected animals were used as control (n = 4).

### *In vivo* treatment for survival analysis

BALB-c/nude mice were engrafted subcutaneously with 5 x 10^6^ NCI-H69 cells in 200 µl HBSS containing 33,3% Matrigel or with 5 x 10^6^ CA20948 cells in 200 µl HBSS. Mice with NCI-H69 tumors (n = 8) were injected when tumor volumes reached 697 ± 256 mm^3^ and mice with CA20948 tumors (n = 9) when tumor reached 318 ± 216 mm^3^. Both groups were compared to vehicle injected counterparts (n = 8 and n = 6, respectively). Tumor volumes were measured three times per week p.i. Mice were sacrificed when tumor volumes reached the humane endpoint of 2000 mm^3^. Tumors were fixed in formalin for 1 day at RT and stored in 70% ethanol until they were embedded in paraffin.

### *In vivo* single photon emission computed topology/magnetic resonance imaging (SPECT/MRI)

On each time-point p.i. SPECT/MRI-scans were performed post mortem on one mouse per group in the radiobiology cohort. SPECT/MRI was performed using a 4-head multipinhole SPECT/MRI system (NanoScan; Mediso Medical Imaging). SPECT images were acquired in 30 min (28 projections; 60 s/projection). Maximum Likelihood Estimation Method image reconstruction was performed with 32 iterations showing full signal recovery. MRI of the mice was done using a T1 gradient echo sequence (repetition time/echo time, 12/2 ms, 1 average) and T2-weighted images were acquired using a and a spin echo sequence (repetition time/echo time, 4,500/41 ms, 4 averages). The other scan parameters included a 70-mm field of view, a 128 × 128 matrix, and a 1-mm slice thickness. The MRI images were used to delineate tumor tissue and were quantified using the scanner software (Nucline, version 3; Mediso Medical Imaging). On the T2-weighted MR images, a region of interest was drawn around the tumor that best showed the tissue boundaries. The total SPECT uptake within the region of interest was divided by the tumor volume to give a volumetric uptake (kBq/mm^3^).

### Pharmacokinetics and dosimetry assessments

The biodistribution data were analyzed to determine the kinetics of the activity in organs and tumors. The measured activity data as a function of time were fitted with single or double exponential curves using the least-square regression method with Graphpad Prism version 5 (graphpad.com). Decisions on to use a single or a double exponential curve was based on the (corrected) Akaike's information criterion 16. The time-activity curves were integrated over time to determine the time-integrated activity coefficients after folding in the lutetium-177 decay function. Absorbed doses in all organs and in the tumor were calculated by using the MIRD equation with the lutetium-177 S-values for a 22 g mouse Moby phantom, kindly provided by Dr Erik Larsson 17.

### Hematoxylin and eosin (H&E) staining

Paraffin tissue sections of 4 µm were cut using a microtome (Microm, 800-1683). Slides were dried overnight at 37 °C. Sections were deparaffinized in xylene and rehydrated in decreasing alcohol concentrations. Sections were then incubated in Harris Hemotoxylin (Sigma-Aldrich, HHs16) for 1 min at RT and subsequently in eosin yellowish solution (AppliChem, 251299) for 1 minute. Slides were then dehydrated in increasing alcohol concentrations and cleared in xylene. The slides were mounted using pertex (Histolab, 00811). Images were procured using a BX40 light microscope (Olympus).

### Antibodies

For immunofluorescent (IF) stainings p53 binding protein 1 (53BP1) (Novus Biologicals, NB100-904; 1:500), phosphorylated histone 2AX (γH2AX) (Millipore, JBW301; 1:250), SST_2_ (Abcam, 134152; 1:100) and Cluster of differentiation 31 (CD31) (Abcam, ab28364; 1:100) primary antibodies were used. Secondary antibodies used are donkey-anti-rabbit IgG Alexa Fluor 594 (Thermo Fisher, A-11078; 1:500) and donkey-anti-mouse IgG Alexa Fluor 488 (Thermo Fisher; A-11005; 1:500). For immunohistochemical stainings (IHC) Ki-67 (Abcam, ab15580; 1:200) was used. Here, the secondary antibody used is Peroxidase donkey-anti-rabbit IgG (Jackson Immunoresearch, 711-035-152; 1:2000).

### Immunofluorescent stainings

Cells were IF stained as previously described 10. Briefly, tissue sections were deparaffinized as described above. Then antigen retrieval was performed by boiling slides for 20 minutes in pH 6 (for cytoplasmic antigens) or pH 9 (for nuclear antigens) antigen retrieval buffer (DAKO) and cooling down to RT. Tissues were permeabilized with PBS+0.5% Triton (PBS(T)) at RT and blocked with 3% BSA in PBS(T) at RT. Sections were incubated with primary antibodies overnight at 4 °C in block buffer and with secondary antibodies for 60 minutes at RT. Mounting was done using Vectashield containing DAPI (Vector labs, H-1200).

### Immunohistochemical stainings

Tissue sections of 4 µm were deparaffinized and rehydrated. Samples were then incubated in 3% H_2_O_2_ (Honeywell, 95299) in methanol (Honeywell, 32213-2.5l) for 20 minutes at RT. Slides were washed with tap water and boiled in pH 6 Antigen Retrieval buffer for 20 minutes. Slides were incubated in blocking solution (PBS 5%BSA), following with the primary antibody in blocking solution overnight at 4 °C and with the secondary antibody in blocking solution for 90 minutes at RT. Slides were washed and incubated for 1 minute in DAB-solution (DAKO, K3468). The slides were dehydrated, cleared and then mounted using Pertex (Histolab, 00811). Images were procured using a BX40 light microscope (Olympus). Quantification was performed in ImageJ by using particle analysis after.

### TUNEL assay

Tissue sections of 4 µm were deparaffinized and rehydrated. TUNEL assay was performed using the In Situ Cell Death Detection Kit, Fluorescein (Roche, 11684795910) according to manufacturer's instructions.

### Confocal microscopy and quantification

53BP1 and γH2AX focus formation was imaged with a Leica SP5 confocal microscope (Leica) using Z-stack acquisition. For tile-scan analyses a LSM700 confocal microscope (Zeiss) was used. Tiles were stitched, thresholded and quantified using ImageJ.

ImageJ was utilized to apply the same local threshold (default for DAPI, MaxEntropy for SST_2_) to all images in order to segment nuclei or quantify DAPI signal and quantify IF signal. Foci were quantified using the Find Maxima function.

### Statistical analyses and mathematical models

Statistical analyses were performed in GraphPad Prism version 8.2.1. Analyses conducted were unpaired Student t-tests or one-way ANOVA followed by Bonferroni's or Tukey's test for multiple comparisons or Browne-Forsythe and Welch posttest for comparisons to control samples. For the correlation between SPECT and *ex vivo* bio distribution data the Pearson correlation was performed. All tests with values of p < 0.05 are assumed significant.

## Results

### Biodistribution of [^177^Lu]Lu-DOTA-TATE shows high radioactive uptake in tumors and kidneys

**Figure 1:**
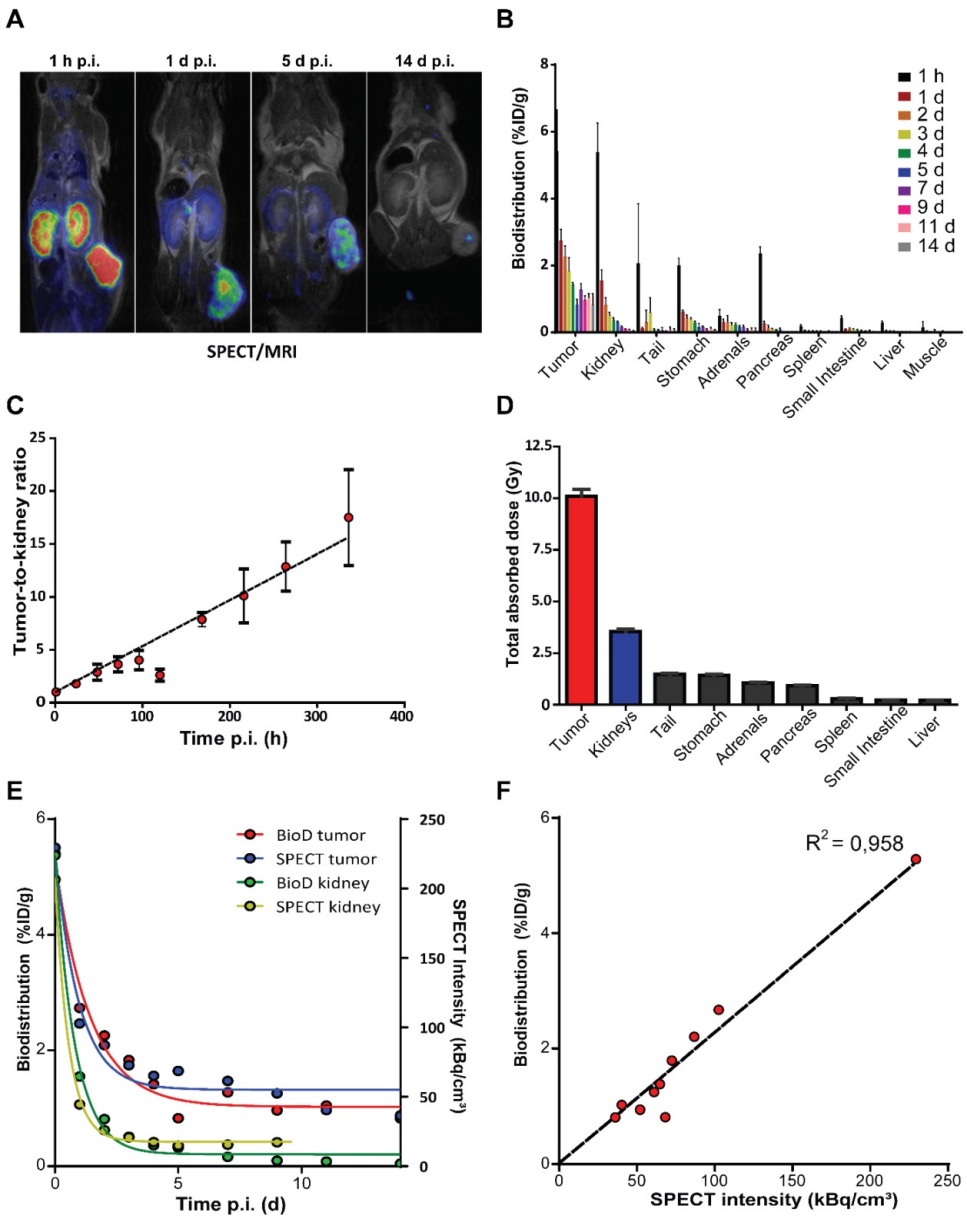
***In vivo* and *ex vivo* biodistribution of [^177^Lu]Lu-DOTA-TATE in NCl-H69 xenografted mice.** (A) SPECT/MRI scans of [^177^Lu]Lu-DOTA-TATE injected mice at different time-points (n = 1). (B) The biodistribution measured *ex vivo* over time in percentage of injected dose per gram of [^177^Lu]Lu-DOTA-TATE in the tumor and healthy organs (n = 4). Error bars indicate standard deviation. (C) The [^177^Lu]Lu-DOTA-TATE uptake tumor-to-kidney ratio over time based on the biodistribution data. Error bars indicate standard deviation. (D) The total absorbed dose of the tumor and healthy organs based on the *ex vivo* bio distribution data. Error bars indicate standard deviation (n = 4). (E) Comparison of the biodistribution (left Y-axis) and SPECT (right Y-axis) measurements of radioactivity in the tumor and kidney. (F) Pearson's correlation of the biodistribution measured *ex vivo* and SPECT measurements of accumulated radioactivity in tumors.

To evaluate the *in vivo* distribution of [^177^Lu]Lu-DOTA-TATE, we performed SPECT imaging and measured radioactivity *ex vivo* in excised organs of NCl-H69 xenografted mice. Accumulation of lutetium-177 was visualized by SPECT starting from 1 h p.i. with the highest accumulated uptakes in the tumor and kidneys (Figure 1A; Figure S1A). The highest level of lutetium-177 retained in the tumors, which could be visualized up to 14 days p.i. by SPECT and γ-counter measurements (Figure 1A-B; Figure S1A). Inferring from the *ex vivo* biodistribution data we found an increasing tumor-to-kidney ratio over time, ranging from 1.01 ± 0.18 to 17.50 ± 4.52 at 1 h and 14 days p.i., respectively (Figure 1C). Furthermore, we have calculated the absorbed dose in all analyzed organs and found substantial absorbed doses received by both tumor and kidney of 10.08 ± 0.35 Gy and 3.5 ± 0.12 Gy, respectively and an absorbed dose ranging from 0.22 to 1.48 Gy for the other organs (Figure 1D; Figure S1B).

When quantifying the level of lutetium-177 on the SPECT scans, we observed a distribution very similar to the measured radioactivity *ex vivo* in the tumors and kidneys (Figure 1E-F; Figure S1C).

### Therapeutic response is induced heterogeneously in the tumors

**Figure 2:**
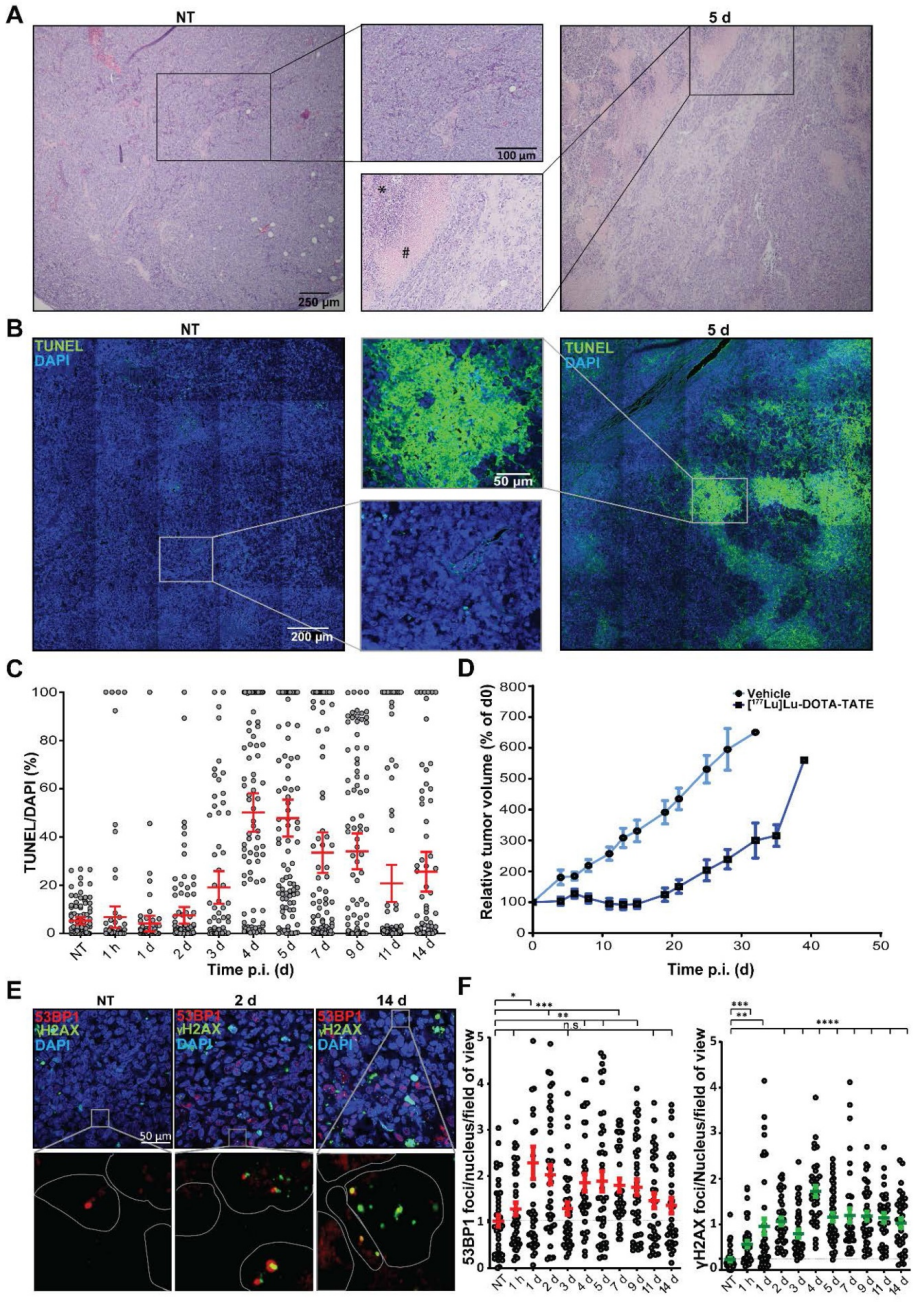
** Histological analysis of [^177^Lu]Lu-DOTA-TATE induced cell death and DNA damage.** (A) Representative H&E images and zoom of NCI-H69 tumors of non-treated (NT) mice and 5 days after [^177^Lu]Lu-DOTA-TATE injection. (B) Representative TUNEL tile-scan images and zoom of NCI-H69 tumors of non-treated mice and 5 days after [^177^Lu]Lu-DOTA-TATE injection. (C) Quantification of the TUNEL signal in DAPI stained cells of 25 fields of view per tumor sample (n = 4). Error bars indicate standard error of the mean. (D) Tumor growth curves of NCI-H69 tumors after vehicle (blue) or [^177^Lu]Lu-DOTA-TATE (red) injection of the mice. Error bars indicate standard error of the mean (vehicle n = 9, [^177^Lu]Lu-DOTA-TATE n = 8). (E) Representative images of DNA damage markers 53BP1 (red) and γH2AX (green) in NCI-H69 tumors of NT mice or 2 or 14 days after [^177^Lu]Lu-DOTA-TATE injection. (F) Quantification of 53BP1 (left panel) and γH2AX (right panel) foci in cells in 5 field of view in NCI-H69 tumors NT mice or at different time points post [^177^Lu]Lu-DOTA-TATE injection. Error bars indicate standard error of the mean.

To investigate the therapy response of the NCl-H69 tumors at the cellular level, we performed histochemical analyses. H&E stainings of the different tumors showed large pyknotic and disintegrating regions starting from 2 days p.i. of [^177^Lu]Lu-DOTA-TATE, indicating induction of cell death. These regions were heterogeneously distributed in the tumors and were diverse in size. Also, these regions became more prominent until day 7 p.i. (Figure 2A; Figure S2).

To further analyze the kinetics and heterogeneity of cell death induction, we performed a TUNEL assay, which stains cells with fragmented DNA, indicating apoptosis (Figure 2B). The level of TUNEL signal started increasing from 3 days p.i. TUNEL signal reached the highest level 4 and 5 days p.i. with an average of 50% and 47% TUNEL positive areas, respectively (Figure 2C). The fraction of TUNEL positive cells then declined to an average of 26% at 14 days after treatment. We observed a large variation in size and distribution of TUNEL positive areas, which was corroborated as a large spread in the quantification (Figure 2C).

Furthermore, we measured the therapeutic effect in an additional group of mice. Tumor growth curves showed that 5 days p.i. of [^177^Lu]Lu-DOTA-TATE NCI-H69 tumors start to decrease in volume to 71% of the starting volume at 14 days p.i., upon which regrowth occurred (Figure 2D).

### DNA double strand breaks are induced in a heterogeneous manner

The therapeutic response of [^177^Lu]Lu-DOTA-TATE can be attributed to induction of DNA damage of which DNA double strand breaks (DSBs) are the most cytotoxic 18. Therefore, we investigated the level of PRRT-induced DSBs and their repair kinetics on the different time-points p.i. 19. IF staining of the DNA damage markers 53BP1 and γH2AX, showed a strong induction DNA damage response (Figure 2E). 53BP1 foci was observed from 1 h p.i. (Figure 2F). A further increase and large spread in the number of foci per cell was observed at 1 and 2 days p.i. to an average of 2.3 ± 2.2 and 2.0 ± 1.3 respectively, after which the number 53BP1 foci declined again, yet remained significantly higher than tumors from vehicle treated animals. We observed a 5-fold increase in the number of γH2AX foci 2 days p.i. to 1.1 ± 0.5 foci per cell per which retained significantly higher than NT controls throughout all time-points. For both markers intra-tumor heterogeneity was observed. Interestingly, 53BP1 and γH2AX foci did not colocalize in all cells.

### The heterogenic therapy response is not explained by variation in proliferation and bioavailability

**Figure 3:**
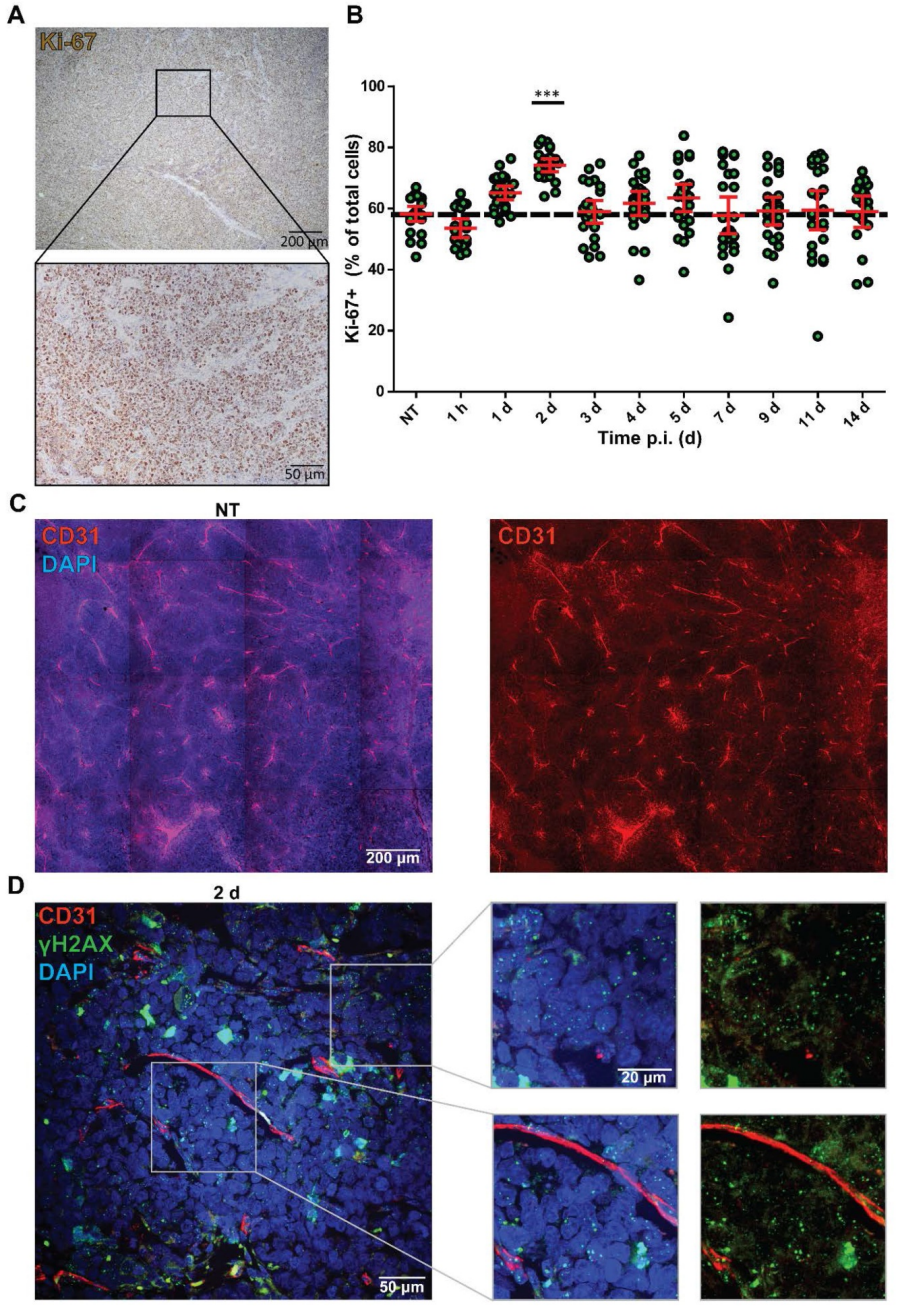
** Analysis of Ki-67 status and vasculature in correlation with DNA damage levels.** (A) Representative image of the Ki-67 staining of NCI-H69 tumors of non-treated mice. (B) Quantification of Ki-67-positive cells over time. ***p < 0.001 compared to NT. Error bars indicate standard error of the mean. (C) Representative tile-scan image of CD31 staining of a NT NCI-H69 tumor. (D) Representative Z-stack image with zooms of CD31 staining (red) and γH2AX staining (green) of NCI-H69 tumors two days post [^177^Lu]Lu-DOTA-TATE injection. Depicted are areas directly next to vessels (lower panel) and further from vessels (upper panel).

Although it is often assumed that cell line-derived tumors have a homogenous composition, we observed a heterogenic intra-tumoral therapy response. Therefore, we investigated whether the distribution of cycling cells could underlie this heterogenic therapy response by measuring Ki-67 expression (Figure 3A). The NCl-H69 tumors showed a homogeneous distribution of cycling cells. Quantification of the fraction of cycling cells in non-apoptotic regions showed 60.96 ± 5.32% cycling cells in all samples regardless of therapy, the only exception being 2 days after treatment, where the level of Ki-67 positive cells was significantly increased to 74.13% (Figure 3B).

Another possibility that can augment therapy response is the bioavailability of a drug to different parts of a tumor. Therefore, we analyzed the vascularization of the tumors by staining for the endothelial cell marker CD31 in NT and tumors 2 d p.i. Here, we observed an equal distribution of blood vessels in the whole tumors (Figure 3C). Moreover, we analyzed the proximity of DNA damage to blood vessels by combining IF stainings of CD31 with γH2AX at 2 days p.i. with [^177^Lu]Lu-DOTA-TATE.

No correlation between presence of CD31-positive vessels and level of induced DNA damage was found in these tumors as γH2AX foci were observed regardless of the distance to blood vessels (Figure 3D).

### DNA damage levels induced by [^177^Lu]Lu-DOTA-TATE are correlated to SST_2_ expression levels

**Figure 4:**
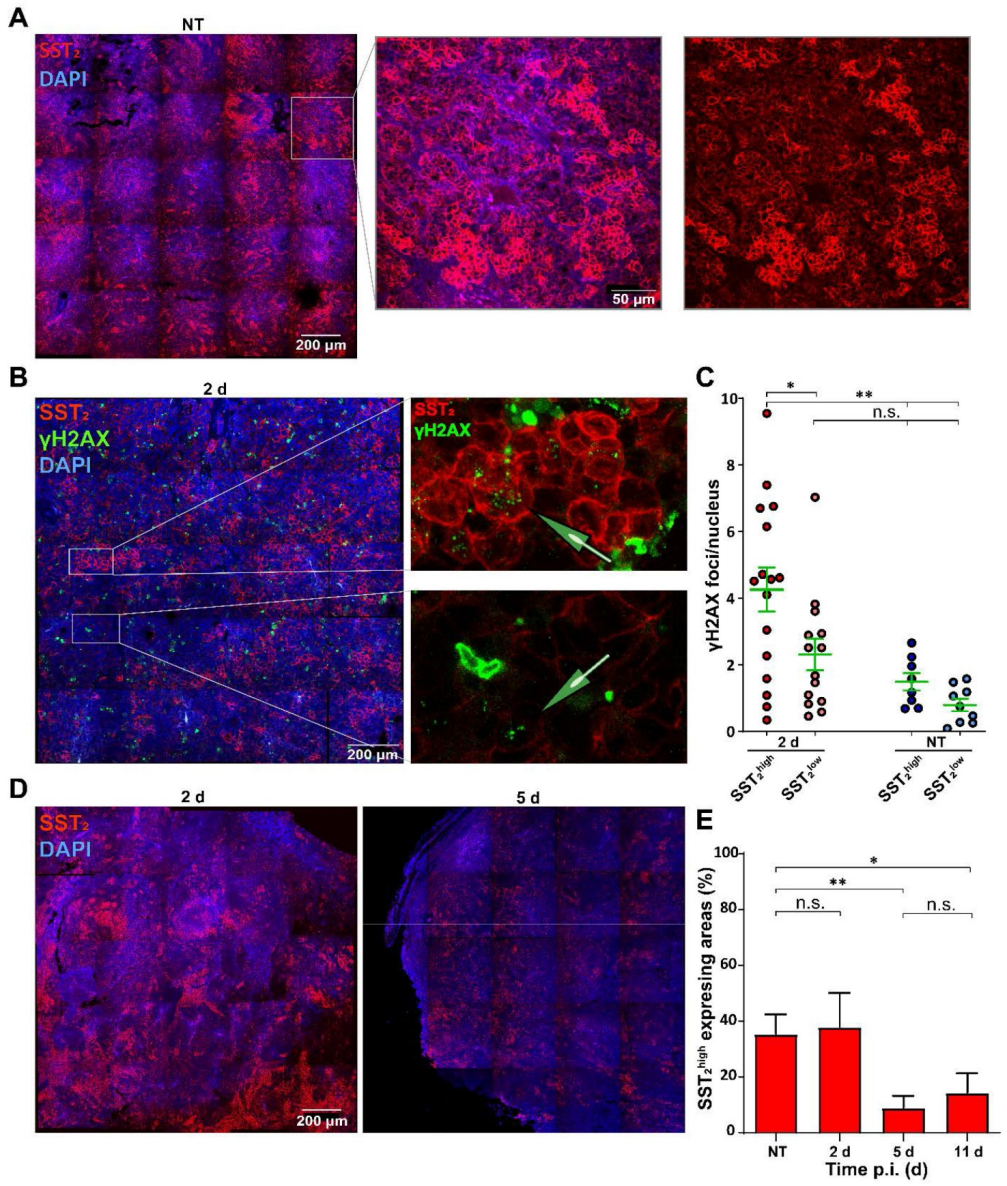
** Analysis of SST_2_ expression levels in correlation with DNA damage.** (A) Representative tile-scan image with zoom of SST_2_ stainings of NCI-H69 tumors of NT mice. (B) Representative tile-scan image with zoom of SST_2_ and γH2AX stainings of NCI-H69 tumors 2 days after [^177^Lu]Lu-DOTA-TATE injection. (C) γH2AX foci quantification in SST_2_^high^ and SST_2_^low^ regions of NT tumors and 2 days p.i. Error bars indicate the standard error of the mean (n = 4) *p < 0.05, **p < 0.01. (D) Representative tile-scan images of SST_2_ stained tumors 2 and 5 days after [^177^Lu]Lu-DOTA-TATE injection. (E) Quantification of the fraction of SST_2_^high^ cells in NCI-H69 tumors of NT mice or at different time points post [^177^Lu]Lu-DOTA-TATE injection. Error bars indicate standard deviation. *p < 0.05, **p < 0.01.

To further investigate the heterogeneous therapy response, we analyzed SST_2_ expression levels. SST_2_ membrane staining was observed in all tumor cells and interestingly, the SST_2_ expression levels differed greatly between clustered NCl-H69 cells within all tumors (Figure 4A).

To investigate whether the differential SST_2_ expression influenced the level of [^177^Lu]Lu-DOTA-TATE induced DNA damage, we performed a co-staining of SST_2_ with γH2AX (Figure 4B). No significant difference in baseline γH2AX foci in cells of SST_2_^high^ and SST_2_^low^ regions was observed in tumors of the non-treated mice. Moreover, SST_2_^low^ cells in the tumors of treated mice showed no significant increase in the number of γH2AX foci compared to SST_2_^low^ cells in tumors of non-treated mice. However, we observed a significant increase in the number of γH2AX foci in SST_2_^high^ cells in tumors from treated mice compared to non-treated SST_2_^high^ regions. Moreover SST_2_^high^ cells showed a significantly higher number of γH2AX foci compared to SST_2_^low^ cells in tumors from [^177^Lu]Lu-DOTA-TATE treated mice (Figure 4C).

Overall the fraction of SST_2_^high^ cells reduced over time (Figure 4D). Tumors from non-treated mice and from 2 days p.i. of [^177^Lu]Lu-DOTA-TATE showed 36% and 38% SST_2_^high^ expressing areas, respectively. Interestingly, tumors at 5 days and 11 days p.i. showed a strong reduction of SST_2_ expression to a fraction of 9% and 14% SST_2_^high^ cells, respectively (Figure 4E).

### SST_2_ expression levels differ between CA20948 and NCI-H69 cells and influence [^177^Lu]Lu-DOTA-TATE uptake

**Figure 5:**
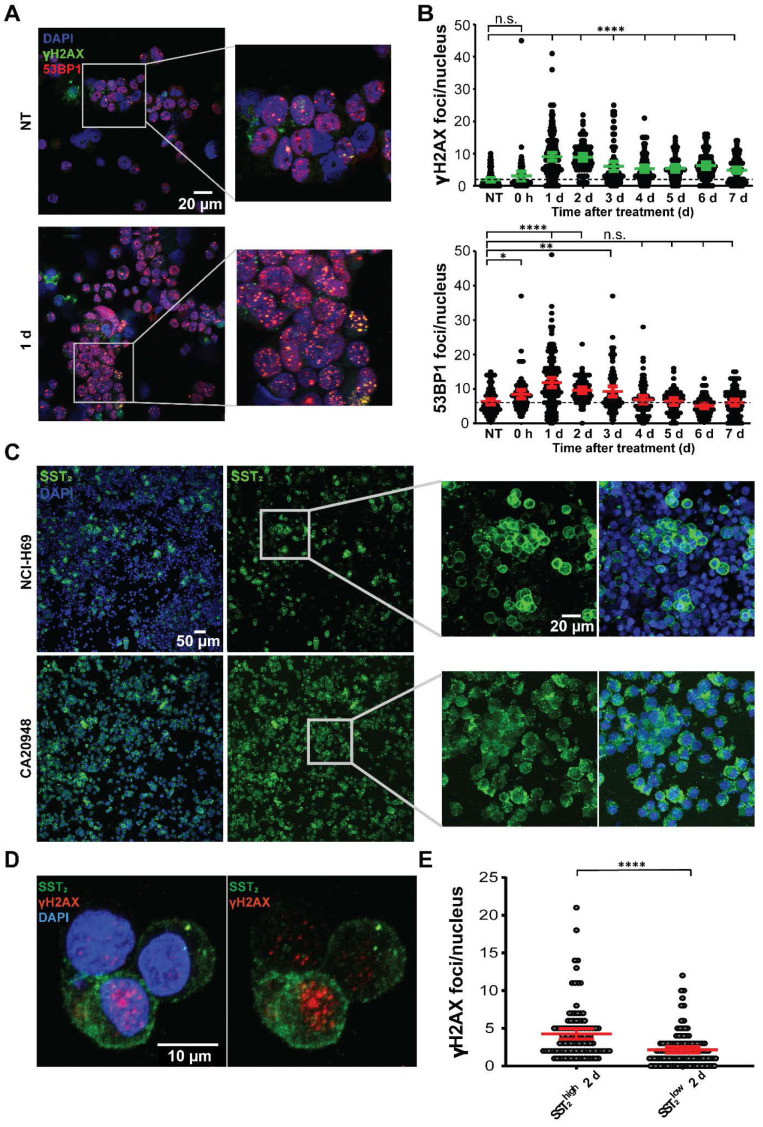
***In vitro* analyses of [^177^Lu]Lu-DOTA-TATE treatment in a time-dependent manner.** (A) Representative images of 53BP1 and γH2AX foci in NCI-H69 cells 1 day after [^177^Lu]Lu-DOTA-TATE incubation and NT cells. (B) Quantification of the number of 53BP1 and γH2AX foci per nucleus in NCI-H69 cells in a time-dependent manner after incubation with [^177^Lu]Lu-DOTA-TATE. Error bars indicate 95% confidence interval, *p < 0.05, **p < 0.01, ****p < 0.0001. Two other independent experiments and average of all three experiments can be found in Figure S3. (C) Representative IF images of SST_2_ expression in NT NCI-H69 cells (upper panels) and CA20948 cells (lower panels). (D) Representative images of SST_2_ expression and γH2AX foci in NCI-H69 cells 2 days after incubation with [^177^Lu]Lu-DOTA-TATE. (E) Quantification of γH2AX foci per nucleus in SST_2_^high^ and SST_2_^low^ regions in NCI-H69 cells 2 days after incubation with [^177^Lu]Lu-DOTA-TATE. Error bars indicate 95% confidence interval. Two other independent experiments and average hereof can be found in Figure S5.

To corroborate the correlation between SST_2_ expression levels, radioactive uptake and DNA damage, we performed [^177^Lu]Lu-DOTA-TATE treatment *in vitro*. We measured a significant increase in the level of 53BP1 and γH2AX foci per cell in NCl-H69 cells at 1 day post treatment compared to non-treated cells, with a large variation in the number of foci per cell (Figure 5A-B; Figure S3). A continued presence of elevated of γH2AX and 53BP1 foci was observed from day 3 until at least day 7 after treatment.

Staining of SST_2_ in NCI-H69 cells again revealed differential receptor expression among the cells (Figure 5C). In order to determine whether this is universal or not for SST_2_ positive models, we analyzed the SST_2_ expression in CA20948 cells, a rat pancreatic cancer cell line which is frequently used in PRRT research 14. In these cells, we observed a much more homogenous distribution of SST_2_ expression levels compared to NCI-H69 (Figure 5C).

Upon [^177^Lu]Lu-DOTA-TATE treatment of both cell lines we observed a 3 fold higher uptake of radioactivity in CA20948 compared to NCI-H69 (Figure S4A). Moreover we observed more SST_2_ internalization directly after incubation with [^177^Lu]Lu-DOTA-TATE in cells that have higher expression of SST_2_ compared to their lower expressing counterparts in both CA20948 and NCI-H69 cells (Figure S4B). In line with this and what was found *in vivo*, we observed that in NCI-H69 cells a significant higher number of γH2AX foci in the SST_2_^high^ cells was measured after [^177^Lu]Lu-DOTA-TATE *in vitro* treatment compared to SST_2_^low^ cells (Figure 5D-E; Figure S5).

### SST_2_ expression levels of CA20948 and NCI-H69 tumors impact [^177^Lu]Lu-DOTA-TATE uptake and therapeutic response

**Figure 6:**
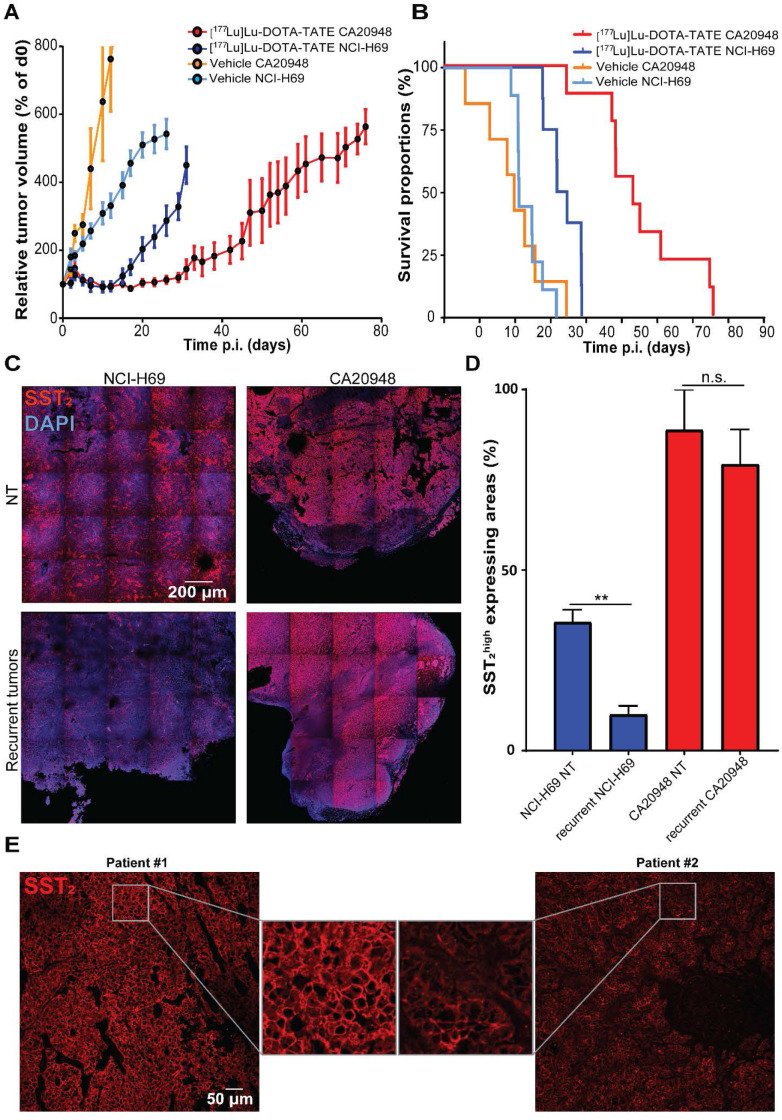
** Survival, SST_2_ expression levels and uptake in different tumor models.** (A) The relative tumor volumes in [^177^Lu]Lu-DOTA-TATE treated mice bearing NCI-H69 or CA20948 tumors compared to vehicle treated controls. Error bars indicate standard error of the mean (NCI-H69 tumors: vehicle n = 9, [^177^Lu]Lu-DOTA-TATE n = 8; CA20948 tumors: vehicle n = 6, [^177^Lu]Lu-DOTA-TATE n = 9). (B) Survival curves of [^177^Lu]Lu-DOTA-TATE treated mice with NCI-H69 and CA20948 tumors belonging to mice in A. (C) Representative tile-scan images of SST_2_ stainings of vehicle treated NCI-H69 and CA20948 tumors and recurrent tumors. (D) Quantification of SST_2_^high^ expression areas in non-treated NCI-H69 and CA20948 tumors and recurrent tumors. Error bars indicate standard error the of mean (n = 4), **p < 0.01. (F) Representative images of SST_2_ staining of two pancreatic NET samples with homogenous (left panel) and heterogeneous (right panel) expression. More IF stained patient samples can be found in Figure S7.

To analyze whether differences in SST_2_ expression levels can influence therapeutic efficacy, we treated CA20948 and NCI-H69 xenografted mice with [^177^Lu]Lu-DOTA-TATE. We observed a stronger therapeutic response in CA20948 tumors compared to the NCI-H69 tumors: CA20948 tumor bearing mice started relapsing at approximately 20 days and NCI-H69 tumor bearing mice at 12 days p.i. (Figure 6A). Moreover, treated CA20948 bearing mice showed a median survival of 54 days compared to 34 days of NCI-H69 bearing mice (Figure 6B). We analyzed the uptake of radioactivity on 2 and 4 days p.i. in the two tumor models. The difference in uptake of radioactivity was 2.5 fold higher in CA20948 tumors 2 days p.i. and 4.7 fold higher 4 days p.i. compared to NCl-H69 tumors (Figure S6). To investigate whether this difference in uptake was caused by receptor expression, we analyzed SST_2_ levels in the two tumor models. In accordance with the uptake levels and therapy efficacy, a higher SST_2_ expression was observed in non-treated CA20948 tumors compared to NCI-H69 tumors. Furthermore, while NCl-H69 tumors had a heterogeneous expression pattern, CA20948 tumors showed a homogeneous SST_2_ expression pattern (Figure 6C top panels). Moreover, when comparing SST_2_ expression levels in recurrent NCI-H69 and CA20948 tumors, we again observed that NCl-H69 tumors exhibit significantly lower expression levels, while CA20948 tumors retained high and homogeneous expression (Figure 6C-D). To determine whether receptor heterogeneity occurred in human NETs, we performed SST_2_ stainings on resected tumor samples from NET patients. Here, we observed differences in expression levels between patients. Furthermore, SST_2_ heterogeneity was observed within these tumors, however to different extents between patient samples (Figure 6E; Figure S7).

## Discussion

In this study we dissected important radiobiological parameters in SST_2_ positive tumors and cells in the context of PRRT. We observed extensive uptake of [^177^Lu]Lu-DOTA-TATE in the tumor which resulted in induction of DNA damage and subsequent cell death, which then mitigates over time. We noted that these therapeutic effects are heterogeneous throughout the NCI-H69 tumors and that this can be, at least in part, attributed to the heterogeneous distribution of the target receptor expression. As SST_2_ expression levels and its intra-tumor distribution differ between the models we used, this might underlie the difference in therapeutic efficacy and the different recurrent tumor phenotypes.

The high observed [^177^Lu]Lu-DOTA-TATE uptake in the tumor and kidneys corroborates previous findings in mice and patients 20. Furthermore, splenic uptake differed between mice and patients 21, 22. [^177^Lu]Lu-DOTA-TATE showed a favorable tumor-to-kidney ratio over time, which is also reflected in the calculated total absorbed dose. Importantly, data acquired with the SPECT/MRI and biodistribution measured* ex vivo*, even though expressed in different dimensions, are in agreement. This confirms an earlier study that analyzed renal uptake of [^177^Lu]Lu-DOTA-TATE in rats using similar platforms 23. The correlation between the two platforms is strong and the feasibility of SPECT as a means of performing dosimetry on absorbed dose in NETs therefore supports (pre-)clinical data 24-26. This could instigate dosimetry studies using SPECT as a means of evaluating biokinetics and intra-tumoral distribution of radioligands in preclinical models, or even patients.

The impact and limiting effects of intra-tumor heterogeneity on therapeutic efficacy are reported in different cancer models 27, 28. Such heterogeneity is observed for many different parameters, such as mutational load 29, epigenetic status 30 and specific protein expression levels 31. This heterogeneity complicates and influences standard of care treatments and emphasizes the need for a better understanding of patient-specific tumor biology and subsequent personalized treatment planning. In line with this, our data showed that upon treatment with a non-curative dose of [^177^Lu]Lu-DOTA-TATE different areas within the tumors suffered extensive DNA damage induction, while other areas remained largely unaffected. We observed a similar pattern for the apoptotic response, which is extensively activated in certain tumor areas, but not in others. In our analyses, recurrent NCI-H69 tumors express the target receptor SST_2_ in a much lesser extent than treatment-naïve tumors, which is in sharp contrast to CA20948 where no decline in SST_2_ expression levels was observed after treatment. This suggests that selective pressure can play an important role when the target is heterogeneously expressed and not when it is homogeneously expressed, which might impact retreatment strategies. From PFS data in patients the response to retreatment regimes seems mitigated compared to the first cycles of PRRT 32, 33. If decreased SST_2_ expression on a cellular level in recurrent lesions plays a role in these mitigated retreatment responses remains to be elucidated. Since it has been described that occasional SST negative lesions also exert a higher grade of dedifferentiation 34 and that SST_2_ heterogeneity between lesions can impact the overall survival of patients 35, follow-up research could focus on the (de)differentiation status of NET cells after PRRT and the impact of this on disease progression.

As our data showed that the distribution of cycling cells is homogenous throughout the tumors, the heterogeneous therapy response is likely not attributed to proliferation. Additionally, blood vessels were well distributed throughout the tumors and no association between the proximity to vessels and DNA damage could be found, thus it is unlikely that bioavailability played a major role in the therapeutic response observed in our study. Although it must be stated that these factors can play major roles in determining therapeutic efficacy and should not be disregarded without care 36, 37. Moreover, we have not analyzed the functionality of the vessels or investigated the perfusion efficiency in these tumors, a factor which has also proven to be of importance to delivery of radioligands 38.

It is described that after the initial 4 cycles of PRRT the majority of patients will present with stable disease, but will eventually show disease progression 5, 39. Patients that present with progressive disease after at least a year and that have responded well to the initial PRRT cycles are eligible for additional cycles of PRRT as it has been shown that retreatment is beneficial in terms of PFS and is often well tolerated 32, 40. A prerequisite for (re)treatment is the presence of positive lesions by [^68^Ga]Ga-DOTA-TATE on a positron-emission-tomography (PET) scan, indicating SST_2_ positive tumors 40, 41. However, information about the SST_2_ expression of single tumor cells within these lesions and their uptake of the radioligand before and after treatment is often unknown. Clinical response to somatostatin analogues is correlated with SST_2_ expression levels. Patients with higher levels of SST_2_ show a significantly higher PFS and longer overall survival after somatostatin analogue treatment compared to patients with lower SST_2_ expression levels 42. Also, it was already shown that [^68^Ga]Ga-DOTA-TATE uptake and SST_2_ expression levels correlate in a preclinical SST_2_ positive xenograft model 43. This was confirmed in a study focused on prostate specific membrane antigen (PSMA), where the investigators showed a correlation between PSMA expression, the fraction of PSMA^high^ cells and therapeutic efficacy with a lutetium-177-labeled PSMA-targeting compound 44. These findings are concomitant with our observation that CA20948 tumors showed higher [^177^Lu]Lu-DOTA-TATE uptake than NCI-H69 tumors, which subsequently led to a significant longer median survival after treatment.

Although PRRT target levels and the fraction of positive tumor cells influence the uptake of radioactivity, this cannot always predict therapeutic efficacy. It has been shown previously that cellular characteristics such as p53 status can strongly influence the sensitivity to radiation in cancer cells 45. Intrinsic differences in radiosensitivity were also demonstrated by analyzing the amount of DSBs after irradiation in a panel of cell lines, which showed that equivalent doses induced varying amounts of DNA damage in different cell lines 46. This suggests that not only the absorbed dose, but also the intrinsic radiosensitivity can influence the therapeutic efficacy of PRRT. Moreover, there is little knowledge as to which radionuclide induces what type of DNA lesions exactly. In the case of lutetium-177 the vast majority of research has been focused on analyzing DSBs by measuring of either 53BP1 or γH2AX foci formation 10, 47, 48. How various other types of DNA damage or the capacity of the target cell to repair these can contribute to sensitivity remains elusive. Interestingly, our data shows that 53BP1 and γH2AX foci do not exclusively overlap, which has been reported before 49. What this means exactly for the underlying DNA damage landscape in these cells will warrant further investigation.

Many different angles for improvement of therapeutic efficacy are currently being investigated as NETs become more prevalent and standard of care treatment is still not curative 6, 39, 50. As we have shown that SST_2_ expression levels can influence cellular therapy effects, it is an interesting concept to stimulate SST_2_ expression to improve therapeutic outcome. Research has shown that certain epigenetic modulators can increase the SST_2_ expression of human pancreatic NET cells 51. Whether this upregulation is limited to tumor cells and not healthy tissues remains to be investigated. Moreover, due to epigenetic heterogeneity within and between NET cells it is still unclear if a tailored epigenetic approach is necessary or possible 52. Furthermore, the options of using different radionuclides, optimizing treatment planning, modulating target expression or applying other combination therapies to improve therapeutic outcomes for patients still warrants further investigation. However, before such options become feasible a better understanding of the radiobiological effects of PRRT is warranted. We believe that our data can contribute to such understanding in various ways. One option would be to use the DNA damage response kinetics data as a touchstone to resolve the ideal time-frame for potential combination therapies, or to determine what time p.i. would be suitable as a read-out for PRRT induced DNA damage. A different option would be to use the SST_2_ heterogeneity in tumors as a strategy for adapted cellular dose-mapping to gain insight in differentially absorbed doses within tumors.

In conclusion, our data deepens the current knowledge on the radiobiological effects of PRRT which can be used to investigate new avenues to improve therapeutic outcome. We describe phenotypic differences between recurrent malignancies of different tumor models and provide evidence for selective pressure in tumors that heterogeneously express the target receptor.

## Supplementary Material

Supplementary figures.

## Abbreviations

[^177^Lu]Lu-DOTA-TATE: [^177^Lu]Lu-DOTA-[Tyr^3^]octreotate
53BP1: P53-binding protein 1
BSA: Bovine serum albumin
CD31: Cluster of differentiation 31
DNA: deoxyribonucleic acid
DSB: Double strand break
IF: Immunofluorescence
MRI: Magnetic resonance imaging
NET: Neuroendocrine tumor
PBS: Phosphate-buffered saline
PFS: Progression-free survival
PRRT: Peptide receptor radionuclide therapy
PSMA: Prostate specific membrane antigen
SPECT: Single photon emission computed tomography
SSB: Single strand break
SST_2_: Somatostatin receptor subtype 2
TUNEL: Terminal deoxynucleotidyl transferase dUTP nick end labeling
γH2AX: Phosphorylated H2A histone family member X
