# Supplementary material for: Inter and intra-tumor somatostatin receptor 2 heterogeneity influences peptide receptor radionuclide therapy response
Source: Theranostics. 2021 Jan 1;11(2):491–505. doi: 10.7150/thno.51215 (PMC7738856; doi:10.7150/thno.51215)
Supplement: Supplementary file 1 — Supplementary figures. [file thnov11p0491s1.pdf]

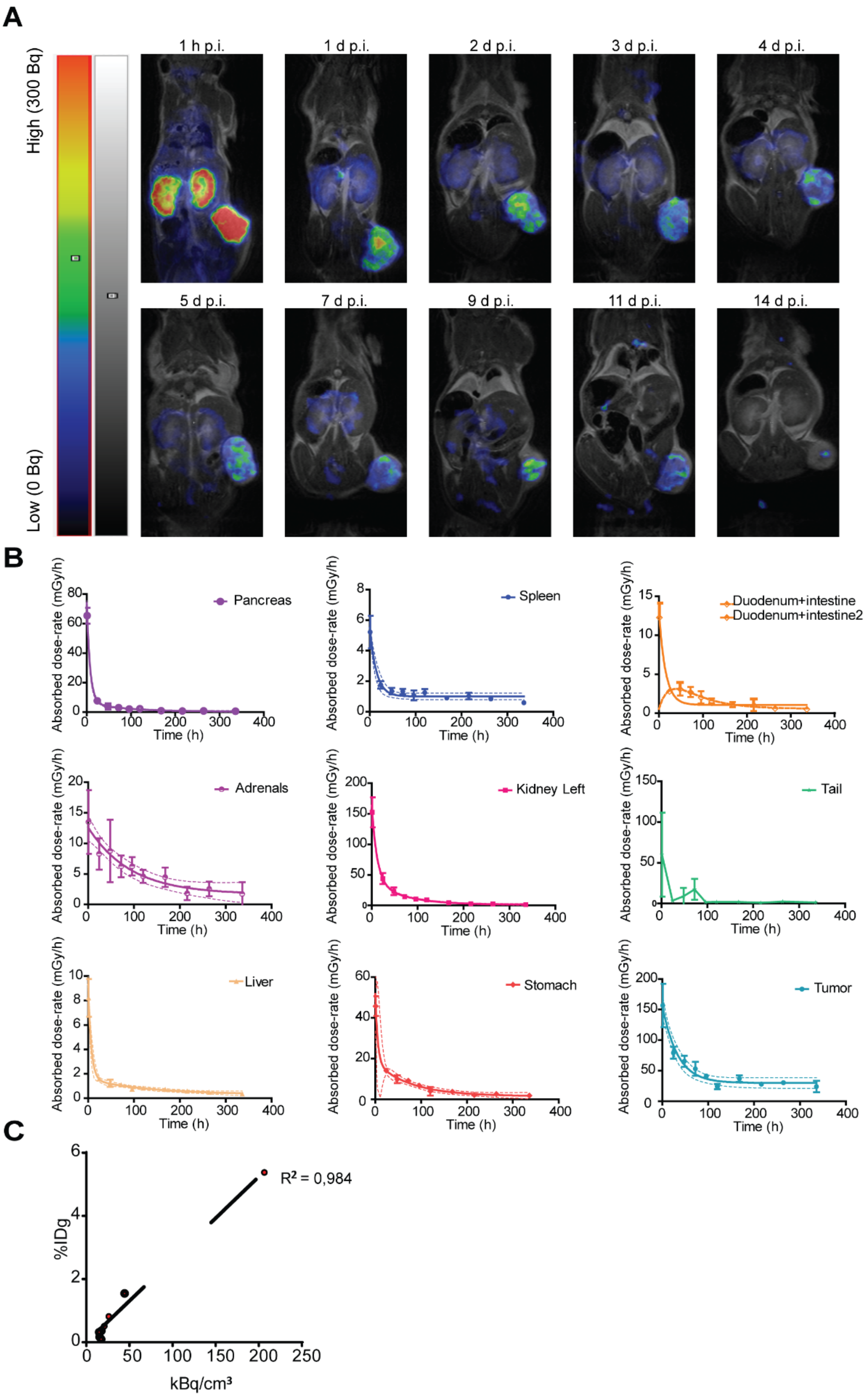

S1. Absorbed dose-rates of analyzed organs and correlation between SPECT and biodistribution. (A) SPECT/MRI images of [^{177}Lu]Lu-DOTA-TATE injected mice at different time points p.i. (B) The absorbed dose-rates of different analyzed organs, depicted in mGy per hour. (C) Pearson's correlation for BioD-SPECT data of kidneys.

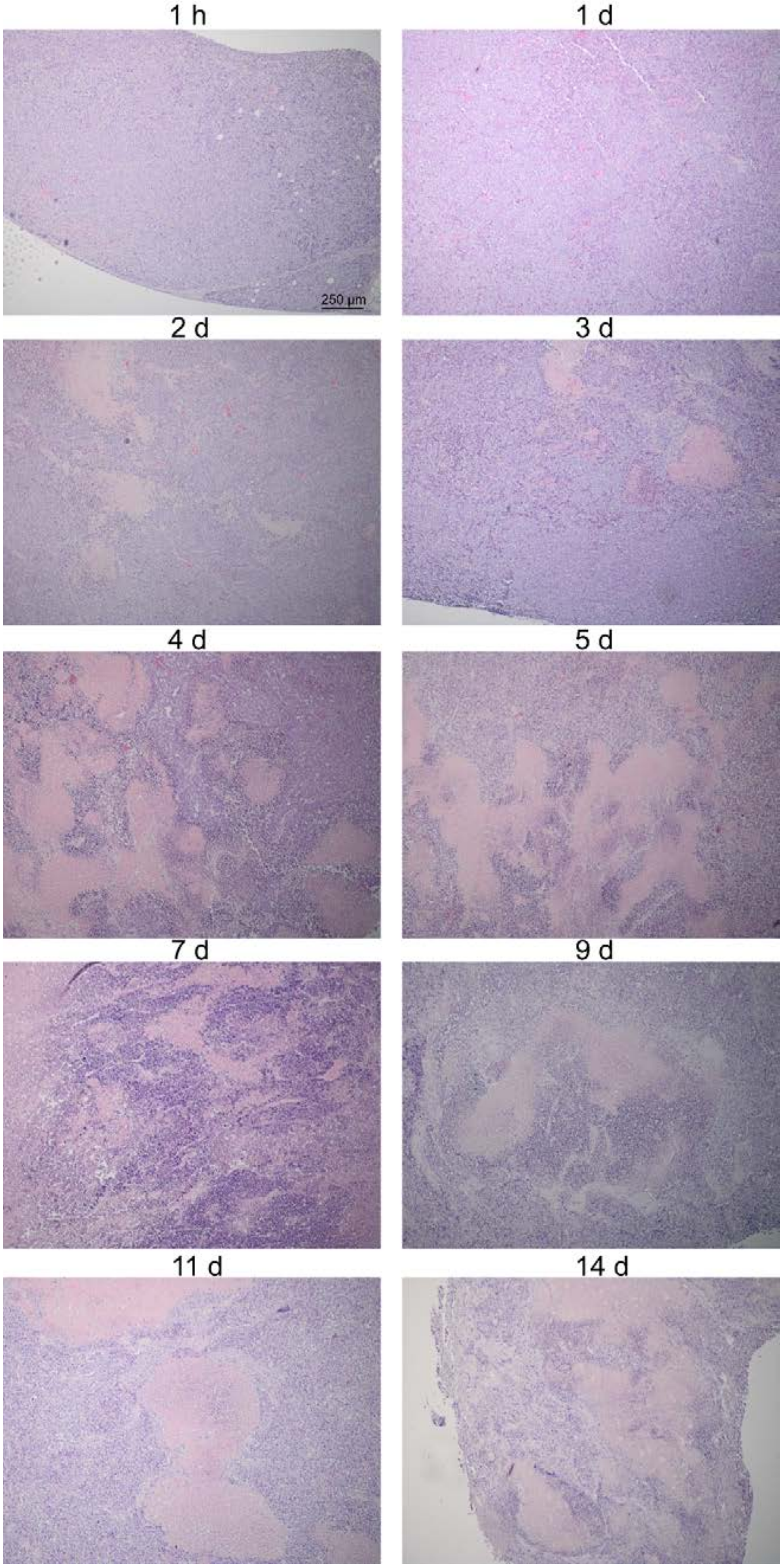

S2. Histopathological overviews of tumors of [177Lu]Lu-DOTA-TATE treated mice. Representative overview images of NCI-H69 tumors on different time points p.i. of [^{177}Lu]Lu-DOTA-TATE.

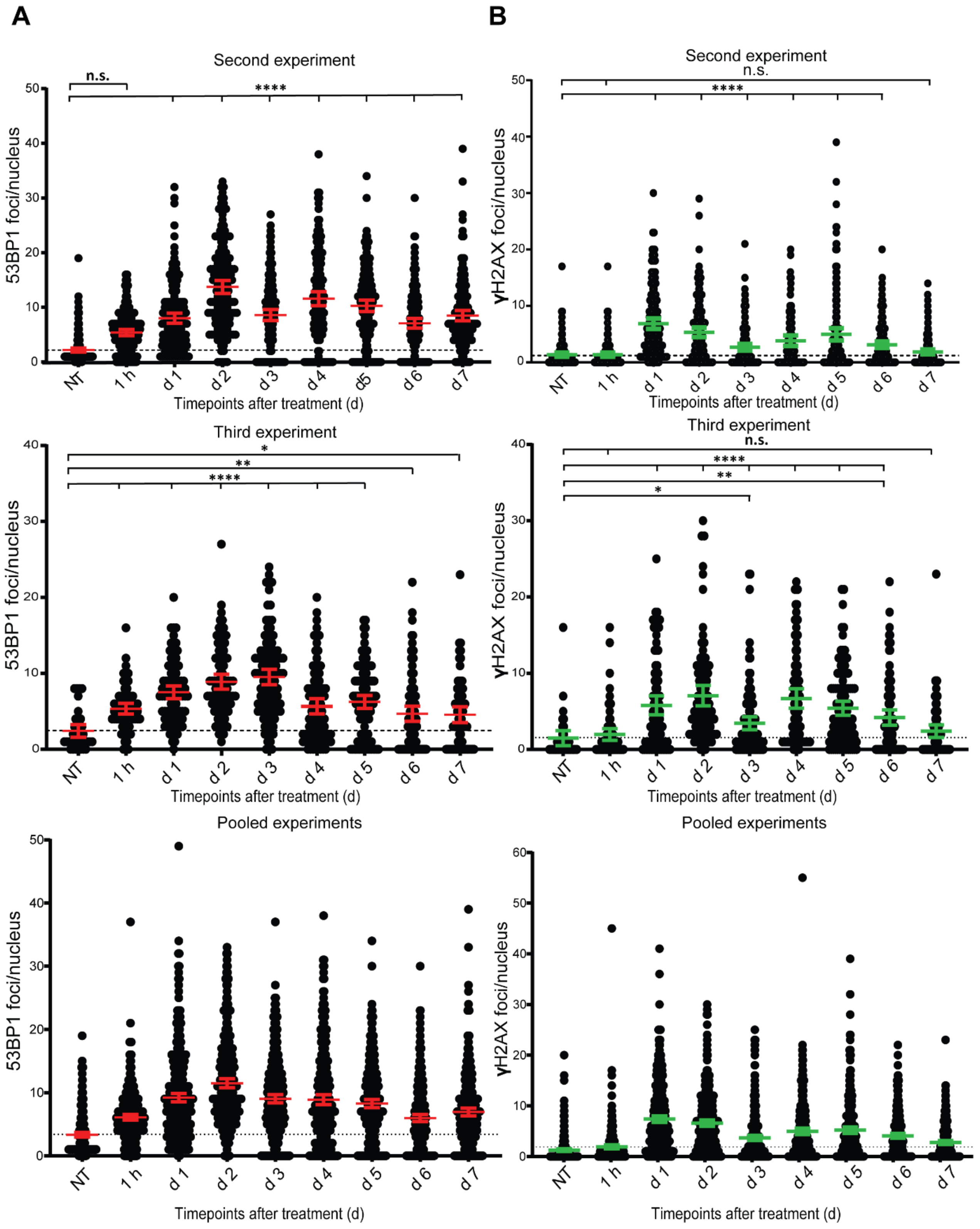

S3. All independent foci quantifications and average in NCI-H69 cells. (A) 53BP1 foci quantification over time in NT versus [^{177}Lu]Lu-DOTA-TATE treated NCI-H69 cells. Error bars indicate 95% confidence interval, *p < 0.05, **p < 0.01, ****p < 0.0001. (B) γH2AX foci quantification over time in non-treated- versus [^{177}Lu]Lu-DOTA-TATE treated NCI-H69 cells. Error bars indicate 95% confidence interval, *p < 0.05, **p < 0.01, ****p < 0.0001.

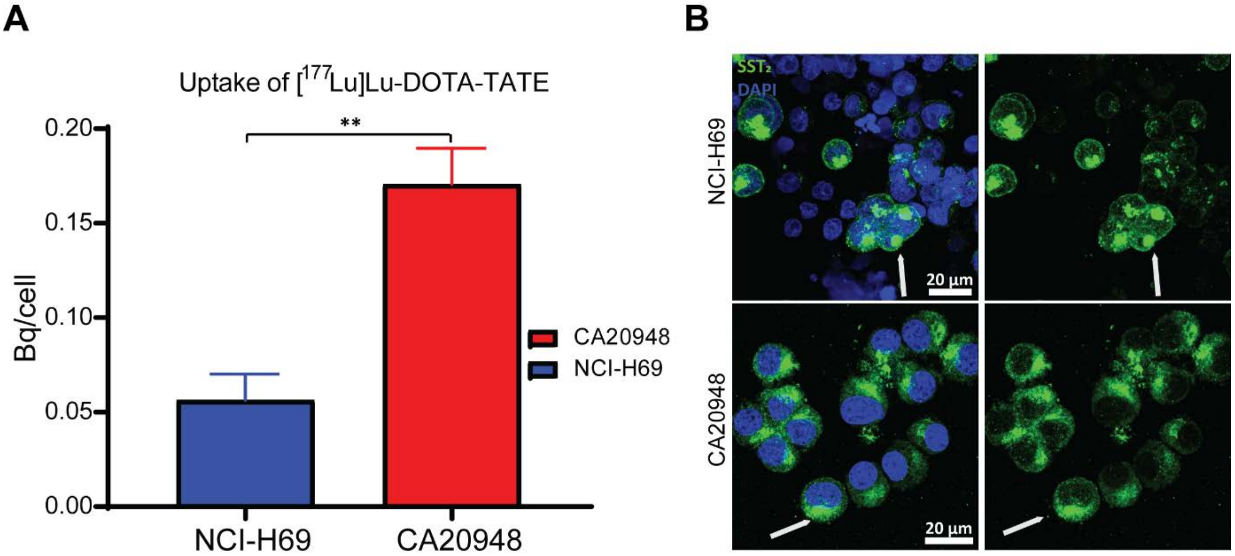

S4. [^{177}Lu]Lu-DOTA-TATE uptake in SST$_2^{high}$ and SST$_2^{low}$ NCI-H69 cells. (A) lutetium-177 uptake measured by γ-counts in NCI-H69 and CA20948 cells after 4 hours of incubation (experiment n = 3). **p < 0.01 (B) Representative IF images of SST$_2$ after [^{177}Lu]Lu-DOTA-TATE treatment. Arrows indicate internalization and accumulation of SST$_2$.

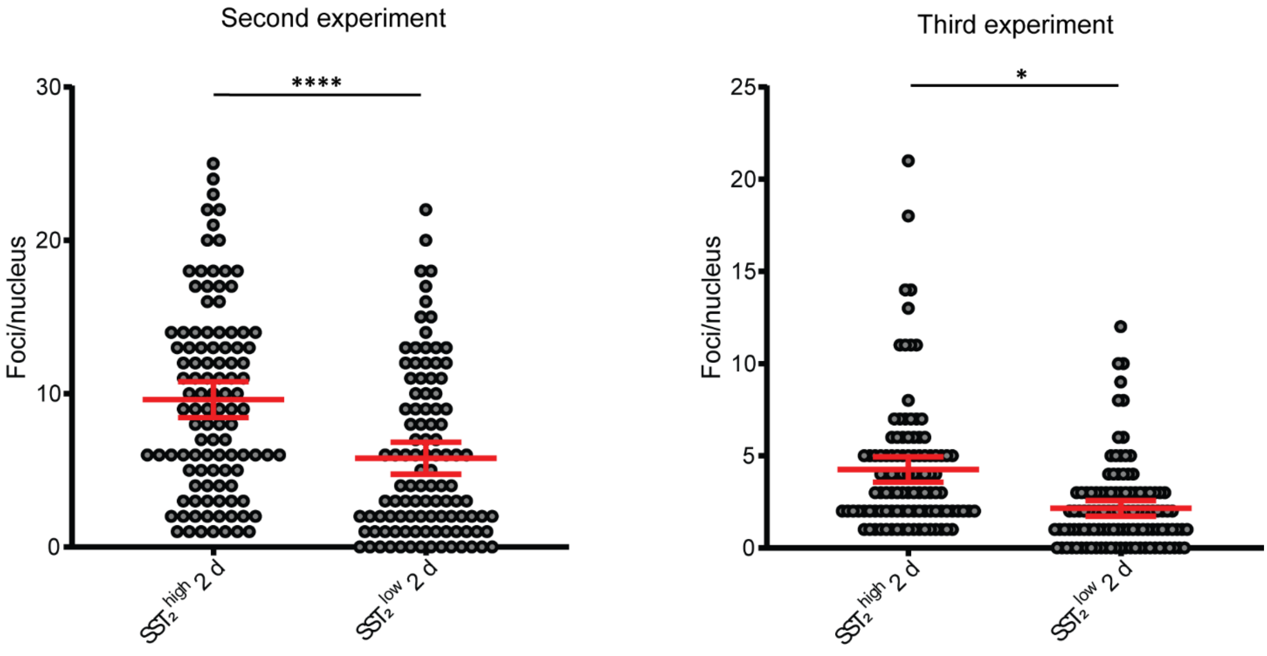

S5. Additional foci quantifications in SST$_2^{high}$ and SST$_2^{low}$ cells. γH2AX foci quantification in SST$_2^{high}$ and SST$_2^{low}$ NCI-H69 cells 2 days after [^{177}Lu]Lu-DOTA-TATE treatment. Error bars indicate 95%CI. *p < 0.05, ****p < 0.0001.

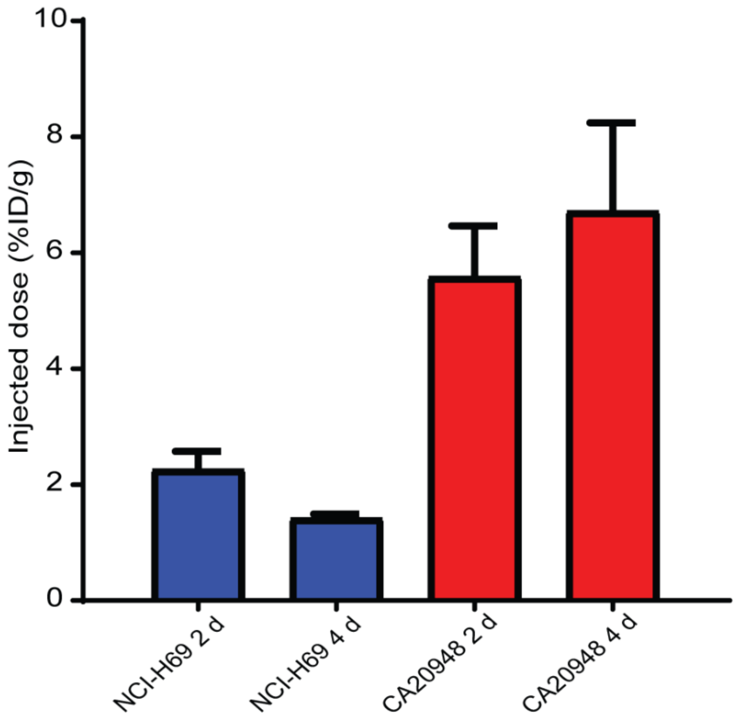

S6. [^{177}Lu]Lu-DOTA-TATE uptake in NCI-H69 and CA20498 tumors measured 2 d and 4 d p.i. Depicted is the percentage of the injected dose per gram (%ID/g) (n = 4). Error bars indicate standard deviation.

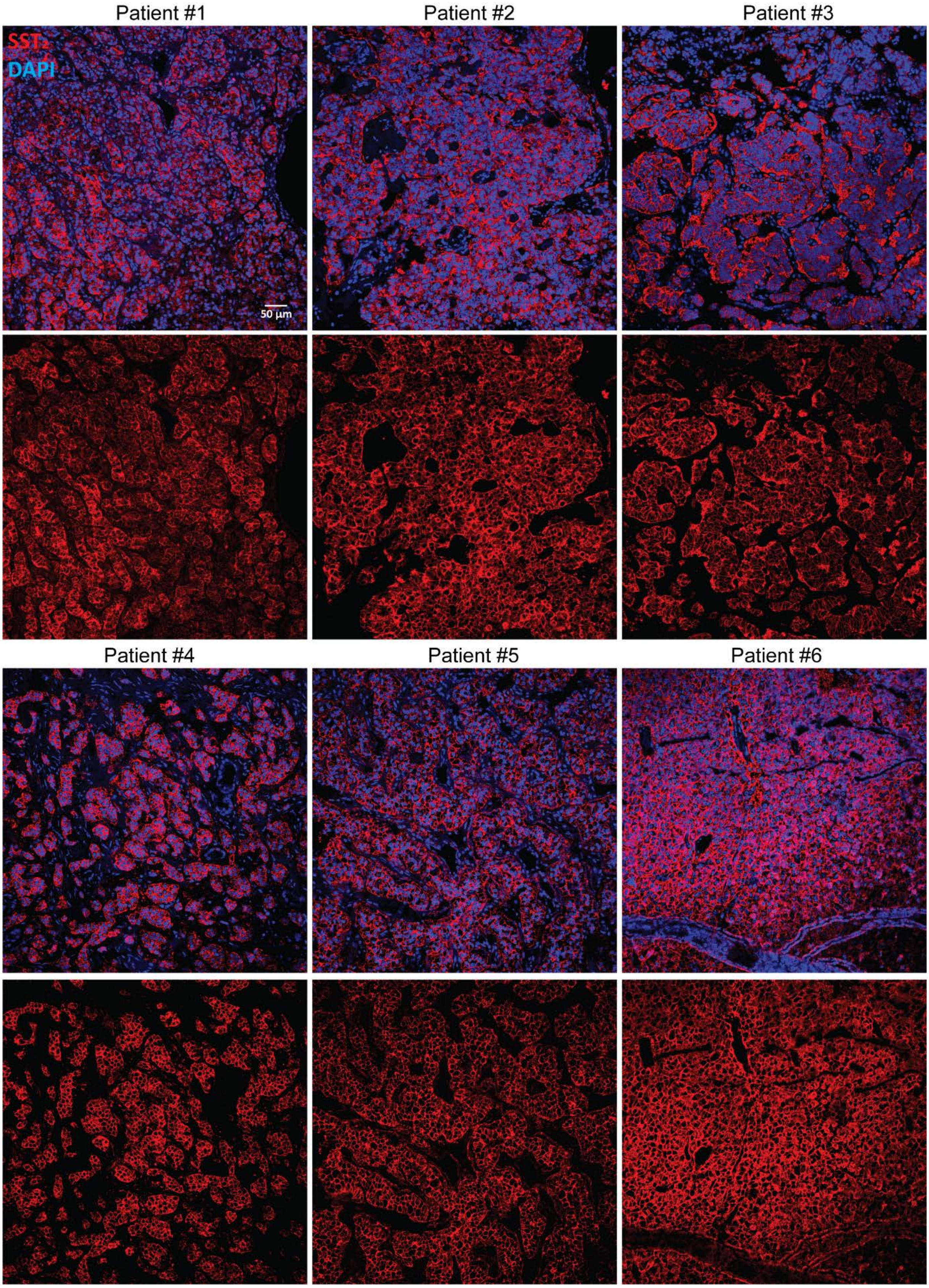

S7. Different degrees of SST_2 heterogeneity in patient NET samples. Representative images of SST_2 stainings on resected pancreatic NETs from different patients.
